# Development of an intervention for the social reintegration of adolescents and young adults affected by cancer

**DOI:** 10.1186/s12889-022-12611-4

**Published:** 2022-02-05

**Authors:** Marie Broholm-Jørgensen, Tine Tjørnhøj-Thomsen, Pia Vivian Pedersen

**Affiliations:** National Institute of Public Health, Research Program on Health and Social Conditions, University of Southern Denmark, Copenhagen, Studiestræde 6, 1455 Copenhagen K, Denmark

**Keywords:** Young cancer patients, Qualitative research, Social reintegration, Adolescents and young adults (AYA), Intervention

## Abstract

**Background:**

In Denmark, around 500 adolescents and young adults (AYAs) aged 15–29 are diagnosed with cancer each year. AYAs affected by cancer constitute a vulnerable group in need of special support in pursuing everyday life as young people. These needs are, however, not currently being adequately met. This study explores the distinctive needs of AYAs aged 15–25 and affected by cancer with the aim of developing and designing an intervention that accommodates these needs and allows AYAs to pursue everyday life following active cancer treatment.

**Methods:**

We combined multiple qualitative methods to conduct six sub-studies: 1) participant observation among support groups for AYAs affected by cancer, 2) field visit at a large Danish hospital, 3) qualitative interviews with AYAs currently or previously diagnosed with cancer, 4) qualitative interviews with practitioners working with young cancer patients or AYAs with chronic conditions, 5) an interactive workshop with practitioners, and 6) an interactive workshop with AYAs. The empirical material was collected between May 2016 and April 2019. The empirical material was read, analysed thematically and coded into the themes; 1) diagnosis and treatment, 2) form of education and 3) age, financial challenges and legal entitlements.

**Results:**

Across the empirical material, we found that AYAs’ cancer experience was heterogeneous. The needs of AYAs differed according to 1) diagnosis and treatment, 2) type of education and 3) age, financial situation and legal entitlements. The findings demonstrate a need for a tailored intervention accommodating the variety of opportunities, requirements and challenges of AYAs with cancer. We propose an intervention consisting of a multidisciplinary team sited at the hospital where the individual AYA receives treatment. The team’s main task will be to maintain AYAs’ social competences and ease their return to everyday life after serious illness by balancing educational requirements with cancer treatment.

**Conclusion:**

Based on the perspectives of practitioners and AYAs affected by cancer, this study outlines an intervention designed as a care pathway in which a multidisciplinary team provides individual and tailored support to AYAs with cancer from the time of diagnosis during and beyond active cancer treatment.

**Supplementary Information:**

The online version contains supplementary material available at 10.1186/s12889-022-12611-4.

## Background

People who survive a cancer diagnosis need help to alleviate physical and psychosocial late complications and adjust to everyday life [1, 2]. This has led to an increased focus on cancer rehabilitation and on providing support for cancer patients reintegrating into everyday life. During recent years, the survival for multiple cancer types in adolescents and young adults (AYAs) has improved [3–5], thereby closing the historical cancer survival improvement gap [4]. In a recent study of survival after cancer in the Nordic countries, Rostgaard et al. found that for the most common AYA diagnostic groups, AYA cancer patients are no longer disadvantaged when compared with children or slightly older adults [5]. However, AYAs with cancer suffer age-specific challenges and are thus faced with age-specific support challenges, which make them particularly vulnerable—especially during the post-treatment phase [6–10].

For AYAs, serious illness may lead to disruptions in everyday activities, school life and social and interpersonal relationships with peers. At the same time, issues of dependence and independence, concerns about physical appearance and existential issues are brought to the fore [9, 11–14]. AYAs face additional challenges because of the intersection of the cancer experience with the developmental tasks associated with this phase of life, such as establishing their identity, developing a positive body image and sexual identity, separating from their parents, increasing their involvement with peers, dating, and beginning to make important life choices and decisions about education [14–18].

A Danish survey (n = 822) of young cancer patients aged 15–29 identified important quality gaps in care and services for AYAs with cancer in Denmark [19]. In particular, post-treatment needs for psychosocial support and needs for support to return to education and work were not met. Overall, 66% of AYA respondents reported needing help to tackle anxiety, sadness or concerns after treatment. Of these, 69% did not receive help in coping with these issues after treatment. Furthermore, 53% reported needing help in maintaining or returning to work and/or education after treatment. Of these, 63% received no help to maintain or return to work and/education after treatment. In addition, the survey identified a range of other post-treatment challenges and unmet needs among AYAs with cancer, for example with issues relating to financial issues and income, or to sex, intimacy and body image [19]. Existing research suggests that the main causes of the poor quality of the post-treatment phase in AYAs with cancer include lack of access to healthcare professionals with specialized knowledge of the AYA population and lack of support in addressing their unique psychosocial and educational needs [16, 20]. Other studies confirm similar unmet needs among AYAs with cancer, indicating additional areas in which they require help in navigating:

1. The multiple transitions through the so-called cancer journey (for example, the various medical transitions, such as transiting from treatment to monitoring for relapse) [21, 22]
2. The various systems involved in the cancer journey, such as the healthcare system, the educational system and local government [10, 16, 17, 23]

Although multidisciplinary teams (MDT) have been set up to address clinical needs in several countries [24]—including Denmark—there is still evidence to suggest that the challenges particular to AYA cancer post-treatment are currently not being addressed adequately [25].

AYAs affected by cancer in Denmark constitute, therefore, a vulnerable group in need of special support beyond active treatment if they are to resume everyday life as young people; however, these needs are currently not adequately met. This study aims to explore the distinctive needs of AYAs, aged 15–25 and affected by cancer, in terms of their social reintegration and return to everyday life during and following treatment. Furthermore, we seek to outline an intervention that accommodates the unmet needs of AYAs in terms of social reintegration. By ‘social reintegration’ we refer to AYAs’ continuation of life and rehabilitation during and after cancer treatment and, hereby, the process of getting back to ‘normalcy’ and everyday life with their family, peers and intimate relations and with their education or work.

## Methods

### Design

We used a sequential study design drawing on multiple qualitative methods [26]. Our overall focus throughout the study was to 1) identify needs and challenges related to AYAs’ cancer journey and to their return to everyday life during and following treatment, and on this basis 2) to develop a relevant and meaningful intervention to facilitate the social reintegration of AYAs with cancer. This required the active involvement of AYAs who had been diagnosed with cancer and of practitioners working within the field of cancer. We focused on AYAs with a current or previous cancer diagnosis and explored the needs and experiences related to their continuation of life during and after cancer treatment. We conducted six sub-studies:

1. Participant observation among support groups for AYAs affected by cancer.
2. A field visit to a Unit for Adolescent Medicine sited at large Danish hospital.
3. Qualitative interviews with AYAs diagnosed, or previously diagnosed, with cancer.
4. Qualitative interviews with practitioners working with young cancer patients or AYAs with chronic conditions.
5. An interactive workshop with practitioners.
6. An interactive workshop with AYAs.

**Fig. 1:**
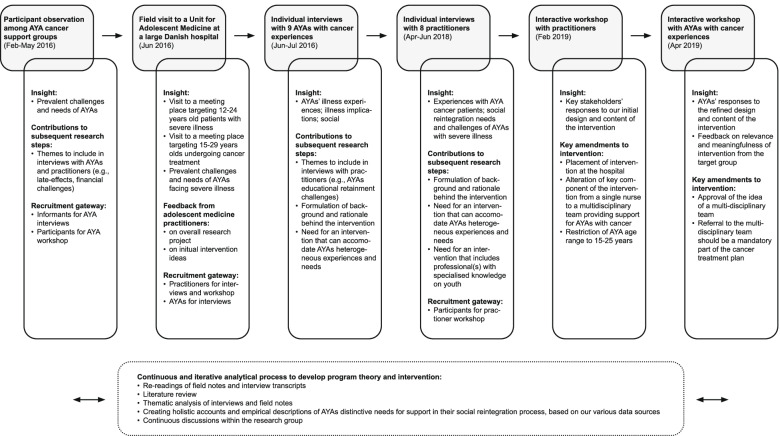
Overview of data collection and processes

We used a sequential design, [26] in which findings derived from the first sub-study informed the design of the next sub-study and so forth. In the development of the intervention, we drew on findings from all sub-studies. We provide an overview of the data collection steps and processes in Fig. 1, which also illustrates how and in what ways the different sub-studies informed each other.

In the following, we present the collection of empirical material in chronological order and elaborate on the contributions of each sub-study.

### Sub-study 1: Participant observation among AYA cancer support groups

AYA cancer support groups (run by the Danish Cancer Society) provided an important means of gaining access to the target group as well as insight into prevalent preoccupations of Danish AYAs with cancer experiences. Through participant observation [27], we gained insight into the practices and social interactions within the support groups and how the participants experienced their everyday lives and the challenges they faced. PVP and a research assistant (Maja Kring Schjørring, who was briefly involved in the research project in its early phases) separately participated in and observed six meetings in five support groups nationwide in Denmark from February to May 2016. The two researchers followed an observation guide, in which a number of points were highlighted for special attention:

- Total number of AYAs present with age, sex, ethnicity, education (if appropriate) and with a description of the appearance of the participating AYAs
- Attention to the setting and the physical location of the meeting (furniture and arrangement of the furniture, smell, noise, etc.) and how the setting framed the social interaction
- Attention to how AYAs made their entrance at the meeting; who initiated the meeting; which topics were brought up; who spoke/did not speak; what interrupted interactions
- Attention to the atmosphere in the room, whether it was tense or relaxed, moments of awkwardness, etc.
- Attention to whether the meeting revolved around particular themes or topics
- Attention to interactions between AYAs concerning their cancer illness (verbal interactions, physical interactions (hugs, showing scars, etc.))
- Attention to the mention of existing initiatives or programmes targeting AYAs with cancer
- Attention to possible existential or ethical issues brought up by AYAs
- Attention to the mention of the attention and awareness given by society at large to cancer and its consequences

The participant observations were not recorded. However, comprehensive field notes were written after each meeting. Meetings took place in the evening and lasted approximately three hours. Most were structured around a central theme, such as sex and sexuality, health and exercise, or existential issues such as fear and death. The themes of the meetings were defined by its voluntary support group leader, all of whom were also young and had cancer experiences, together with the support group members. Typically, the support group leader had invited a guest, such as a priest, sexologist or oncologist, who gave an introductory talk and headed the subsequent discussion. Usually, the participants had dinner together before discussing the evening’s theme. The researcher’s role was primarily as a participant observer [28]. That is, we only participated actively in the sense that we had informal talks with the participants, ate dinner with them and made a short presentation of ourselves and of the research project (approximately 10 min). However, during the discussion of the evening’s theme, we played a role as passive observers to allow the AYAs to share their experiences and feelings.

### Sub-study 2: Field visit to a large Danish hospital

In June 2016, PVP and two colleagues (Maja Kring Schjørring and Morten Hulvej Rod; researchers who were involved in the research project in its early phases) conducted a field visit to a Unit for Adolescent Medicine at a large Danish hospital. The visit had five aims:

1. To visit a hospital meeting place targeting young people (aged 12–24 years) with severe illness
2. To visit a hospital meeting place targeting young people (aged 15–29 years) with cancer
3. To learn more from practitioners working within the field about prevalent challenges and needs of AYAs facing severe illness
4. To get practitioners’ feedback on the research project
5. To discuss initial intervention ideas with practitioners working within adolescent medicine

We met with a chief physician who headed the unit, with a PhD student working within the unit and with a nurse who, as youth coordinator, ran the meeting place for AYAs with cancer. In 2018, we interviewed the nurse as part of the practitioner interviews, and in 2019 both the chief physician and the nurse participated in the practitioner workshop. In addition, both the nurse and chief physician became important gatekeepers. They helped us recruit both AYA informants and other practitioner informants, either directly by providing us with names and telephone numbers or indirectly by sharing information about the research project by e-mail or in closed Facebook groups.

During and after the visit, PVP wrote comprehensive field notes.

### Sub-study 3: Qualitative interviews with AYAs

In June and July 2016, PVP conducted semi-structured interviews [29] with nine young people diagnosed with or having previously had cancer (see Table 1).

**Table 1:** Interviews with adolescents and young adults

ID	Sex	Age	Diagnosis	Age at diagnosis	Stage of illness at time of interview	Education/employment at time of interview
1	F	20–29	Lymphoma	20–24	Fully treated	Student at a university-level institution
2	F	20–29	Lymphoma	20–24	Fully treated	Newly graduated from a professional bachelor course
3	F	20–29	Breast cancer	20–24	Under treatment	Upper secondary student
4	F	10–19	Leukaemia	10–14	Under treatment	Primary and lower secondary school student
5	F	20–29	Breast cancer	20–24	Fully treated	About to begin a shorter course of higher education (1½-2½ years of study)
6	F	20–29	Osteosarcoma	15–19	Fully treated	Medical student
7	M	20–29	Lymphoma	20–24	Fully treated	Unemployed, has completed a vocational upper secondary course
8	M	10–19	Leukaemia	15–19	Fully treated	Upper secondary student
9	M	30–39	Leukaemia	5–10, 15–19 and 20–24	Fully treated	Student at a university-level institution

The individual interviews provided in-depth knowledge about AYAs’ illness experiences, their experiences of late complications, the implications of the illness for their social lives, as well as the most pressing needs regarding social reintegration as perceived by AYAs themselves.

Our overall inclusion criteria were: age (15–25 years old), and current or previous cancer illness. However, recruitment of participants proved more difficult than expected. These recruitment challenges were primarily related to the ongoing illness and treatment of AYAs or to late complications. When approached (either physically or online), some AYAs immediately declined to participate in our study, because they felt they were too weak. Others initially accepted the invitation to participate but ended up withdrawing from the study or stopped responding to our calls. A 24-year-old woman, who had recently resumed her job after treatment for breast cancer, wanted to participate by answering the questions in the interview guide in writing, because she felt that participating in an interview would be too overwhelming for her. However, we never received her answers. These recruitment challenges resulted in an expansion of our age criteria (we included a 30-year-old informant) and required us to use a number of different channels to recruit participants. We recruited four informants in connection with the participant observations of various AYA cancer support groups. Furthermore, we recruited three informants through closed Facebook pages of an AYA cancer support group and from a community for young cancer patients undergoing treatment at a large hospital. We accessed these Facebook pages with the help of a support group leader and a nurse. These gatekeepers helped us by sharing information about our research project with the Facebook group members, and the three informants then texted or called PVP directly to arrange an interview date. Finally, we recruited two informants through a nurse and youth coordinator working exclusively with young cancer patients at a large hospital.

We carried out four face-to-face interviews and five telephone interviews at the informants’ own request.

Telephone interviews have proved to be a useful tool to reach groups that were difficult to access and to obtain sensitive information [30]. Participants’ reasons for this request were both practical and related to their lack of energy. For some, a telephone interview was easier to fit into their busy schedule with school, work, friends and leisure activities, and for others, who were still undergoing treatment or who experienced severe late complications, a telephone interview felt less intrusive and less exhausting.

Based on repeated readings and an initial thematic analysis of the participant observation field notes and of the field visit notes, PVP and TITT developed an interview guide for the AYA interviews. The participant observations as well as the field visit provided general insight into issues preoccupying AYAs affected by cancer and gave rise to themes such as: the dilemma of having been declared cancer-free but not feeling well or fully recovered; the need for psychological support to tackle late complications; the need for increased and improved support to handle financial and administrative issues in the aftermath of cancer; and barriers to recovery related to communicating with public authorities about rights and legislative issues. We included these themes in the interview guides used in the subsequent interviews with both AYAs and practitioners. In this way, we used findings from the participant observations and the field visit to develop and inform the interview guides and the intervention. During the interviews, PVP did not adhere slavishly to the interview guide but instead used it as a flexible support tool to ensure that all major themes were covered, if relevant. Following the first couple of interviews, PVP revisited the interview guide but made no alterations.

Themes covered in the interviews included (see Additional file 1 for the full set of questions for interviews with AYAs diagnosed with, or having previously had, cancer):

- Course and progress of cancer illness and treatment
- Reactions to getting cancer, both own and from social network
- Implications of illness for their social relations (family, friends, intimate partners, etc.)
- Thoughts about and/or experiences of returning to everyday life following treatment
- Thoughts about and/or experiences of returning to school/education following treatment
- Experiences of physical, bodily and mental side-effects and late complications
- Use of social media to communicate about their illness
- Their need for help and support during the course of illness, following treatment and in the aftermath of cancer
- Future and life plans

All interviews with AYAs were audio recorded and transcribed verbatim.

### Sub-study 4: Qualitative interviews with practitioners

From April to June 2018, PVP and MBJ conducted semi-structured interviews with eight practitioners (see Table 2) working within the field of cancer or AYAs with chronic conditions. Of these, six were face-to-face interviews and two were telephone interviews, at the informants’ request. Through these interviews, we gained insight into practitioners’ experiences working with young cancer patients and the challenges involved in supporting the social reintegration of AYAs.

**Table 2:** Interviews with practitioners

ID	Sex	Position	Education
1	M	Child and youth worker at hospital.	Bachelor in Social Education
2	M	Psychologist and project coordinator at hospital.	Psychologist
3	F	Youth coordinator at a haematological clinic. Coordinator of a meeting place targeting young cancer patients undergoing treatment.	Nurse
4	F	Primary school teacher at hospital	Teacher
5	F	Nurse at a paediatric oncological ward. Youth ambassador.	Nurse
6	F	Retired. Former head of medical youth ward. Researcher.	Nurse
7	M	Chief physician on a haematological ward.	Medical doctor
8	F	Chief physician on an oncological ward.	Medical doctor

Our inclusion criteria were professional experience of working with AYAs with cancer or, more generally, of working with AYAs with severe or chronic illnesses, since they may experience challenges during and following treatment similar to those of AYAs with cancer. As shown in Table 2, some of the practitioners interviewed worked exclusively within oncology, whereas others worked in units for adolescent medicine, serving not only AYAs with cancer but AYAs with other severe and/or chronic illnesses. Thus, all practitioners interviewed had worked with AYAs with cancer, but some also had broader adolescent medical experience.

We interviewed the nurse acting as youth coordinator whom we met on the field visit in 2016, and she helped us recruit another nurse. In addition, the head of the Unit of Adolescent Medicine, whom we had also met on the field visit, helped us recruit several practitioners both within and outside the hospital. A social worker of children and young people whom we interviewed helped us recruit a teacher working at another hospital.

MBJ and PVP developed the interview guide for the interviews with practitioners, drawing on findings from both the observations, the AYA interviews and the field visit. For example, we included questions about the challenges related to educational retainment and the educational institutions’ handling of AYAs’ illness and absenteeism due to treatment. Topics covered in the interviews included (see Additional file 2 for the full set of questions for interviews with practitioners working within the field of cancer or AYAs with chronic conditions):

- Their own professional experience, AYA cancer experience, work description
- Description of the AYA initiative and target group that the informant is working with
- Views on challenges and needs among AYAs with cancer
- Views on AYAs’ challenges in returning to education following treatment
- Views and ideas on future initiatives targeting AYAs with cancer, suggestions and ideas for a future intervention

All interviews with practitioners were audio recorded and transcribed verbatim.

### Analytical steps in exploring AYAs distinctive needs

The aim of this study was two-fold: 1) to explore the distinctive needs of AYAs with cancer in terms of their social reintegration, and 2) to outline an intervention accommodating the unmet needs of AYAs with cancer in terms of social reintegration. Both the exploration of AYAs distinctive needs and the development of the intervention was a continuous, iterative analytical process throughout the research period.

At this point in the research period, the empirical material consisted of field notes from the observations and field visit as well as interview transcripts with both AYAs and practitioners. PVP and MBJ read and re-read all the empirical material and by means of cross-sectional indexing [28], we began exploring and identifying the distinctive needs of AYAs affected by cancer across the empirical material. Through this analytical process, it became evident that the AYA cancer experience was heterogeneous and that their needs differed according to both disease-related issues and issues related to their general life situation.

From here, we continued by collating all material and by organising the material into empirical descriptions of AYAs’ distinct and heterogeneous needs in terms of social reintegration. In this process, we created holistic accounts of the experiences and needs of AYAs affected by cancer by combining our various data sources and by directing our attention to all aspects of AYAs’ lives. Such ‘contextual’ or ‘holistic’ data organisation is useful when attempting to understand interwoven parts of a data set relating to complex social processes [28, 31]. These holistic accounts of AYAs’ distinctive needs were important because they formed the background and rationale behind the intervention; that is, they were the empirical findings that substantiated the outline and content of the intervention. We identified the following three issues shaping whether, how and to what extent AYAs needed support during and after cancer treatment:

- Diagnosis and treatment.
- Type of education and balancing educational requirements and cancer treatment.
- Age, financial challenges and legal entitlements.

We present and describe these three themes in the Results section.

### Analytical steps in the development of the intervention

As mentioned, the development of the intervention was a continuous, iterative analytical process throughout the research period. That is, initial intervention ideas and outlines already emerged following the participant observations and the field visit, indicating a distinct need among AYAs with cancer for an intervention consisting of a key professional (e.g. a nurse) providing very tangible help for AYAs, for example in maintaining contact with their educational institution and/or their local government.

The analytical process of identifying AYAs distinctive needs (described above) confirmed and strengthened further our initial outline of the intervention. Following completion of the practitioner interviews, the analytical process of developing the intervention intensified. We engaged in empirical descriptions of the core components of the intervention, again based on our entire empirical material. We specified and empirically substantiated the different competencies that the key professional in the intervention should possess and the various forms of support/help that proved to be prevalent in the empirical material and could be provided by this professional. The interviews with practitioners pointed towards the importance of basing the intervention on professional(s) with specialist youth knowledge to address the individual and very heterogeneous needs of AYAs affected by cancer. For example, several practitioners stressed that the professionals engaged in the intervention should be experienced users of the HEEADSSS psychosocial interview model for adolescents, focusing on assessment of the Home environment, Education and employment, Eating, Peer-related Activities, Drugs, Sexuality, Suicide/depression, and Safety from injury and violence [32].

In our initial intervention design, which we presented at the practitioner workshop (described below), the intervention was directed by a nurse with specialist knowledge of young people and their lives, who would undertake three key elements of the intervention, covering needs for support identified in our empirical material: 1) having conversations with AYAs; 2) ensuring continuous, stable contact with AYAs’ educational institutions; and 3) providing concrete, practical help to AYAs. In this initial intervention design, we targeted the intervention at AYAs aged 15–30 years. We used the outputs from the two workshops to adjust and refine the design of the intervention [33]. We specify these refinements in the following.

### Sub-study 5: Interactive workshop with practitioners

In February 2019, PVP and MBJ conducted an interactive workshop with seven practitioners working within the field of youth cancer or chronic conditions among young people. Present were three nurses, a schoolteacher, a chief physician, a researcher and a project leader from the Danish Cancer Society. We knew six of the participants beforehand, either through their participation in the practitioner interviews or through our field visit at a hospital. We recruited a seventh participant, who headed the Danish Cancer Society’s section for AYA cancer and the organizational setup behind AYA cancer support groups nationwide. The workshop took place at our research institution and lasted three hours (from 4.30 pm to 7.30 pm). The participants had dinner with the researchers during the workshop. The purpose of the workshop was to obtain key stakeholders’ responses to the initial design and content of the intervention and on this basis refine the intervention to make it relevant and meaningful in a youth cancer context. Prior to the workshop, we had planned to do a ‘Speed Boat activity’, derived from Pavelin, Pundir & Cham [34], to stimulate the discussion and identify possible alterations or improvements to the intervention. However, in practice, the participants themselves quickly started a vivid discussion of the intervention, so we chose not to complete the exercise and instead allocated more time for thorough discussion.

We audio-recorded the practitioner workshop and took comprehensive notes.

The practitioner workshop contributed three major alterations and refinements to the intervention design. First, the practitioners identified the hospital as an important setting for the intervention, because all AYAs received treatment at a hospital and hence were used to coming there. This finding was supported by existing literature pointing to the importance of communication and coordination between the hospital, home and educational institution to ensure a smooth social reintegration [35]. Second, the practitioners suggested that we lead the intervention using a multi-disciplinary team instead of a single professional in recognition of the number, diversity and range of the tasks to be performed. Third, the practitioners suggested that we reduce the age range from 15–30 years to 15–25 years, while still acknowledging the breadth and differences in life situations within this age group.

### Sub-study 6: Interactive workshop with AYAs

In April 2019, we presented an adjusted design of the intervention to an AYA cancer support group. In the adjusted design, the intervention consisted of a multi-disciplinary team (and not a single professional) providing support for AYAs aged 15–25 years.

Recruiting AYAs for the workshop proved difficult. Initially, we had planned for the workshop to take place at our research institution and had contacted all AYA support groups nationwide and asked them to share information about the workshop with their members, either by e-mail or in their closed Facebook groups. However, we did not receive any registrations. We therefore contacted some of the support groups again and asked if we could do the workshop within their facilities and at a date when they had already planned a support group meeting. One support group accepted; the remainder declined because their meetings had already been planned.

In all, six women and one man aged between 19 and 34 were present. The workshop took place within the support group’s facilities and lasted approximately two hours (from 6 pm to 8 pm). Given the time of the day and to help create a relaxed atmosphere, we chose to bring dinner for everyone. The workshop aimed to obtain the participants’ comments and perspectives to refine the intervention even further and make it relevant to AYAs affected by cancer. We presented our intervention programme theory and asked the participants to provide their immediate thoughts and comments on whether they could have benefitted from such an intervention on their own illness pathways.

The AYA workshop was not audio-recorded at the request of some of the participants. Instead, we took comprehensive notes.

At the AYA workshop, the refined version of the intervention, based on a multi-disciplinary team rather than a single professional, received much appreciation. Additionally, the AYA workshop contributed another important refinement of the intervention, namely, designing the intervention as an inherent part of the cancer treatment plan and as something all AYAs diagnosed with cancer are referred to.

### Ethical considerations

The study was approved by the Danish Data Protection Agency and did not require further ethical approval by the Danish National Committee on Health Research Ethics. Written or oral consent was obtained from each study participant prior to interview and participant observations and all informants were informed of the ethical principles involved regarding confidentiality and anonymity, including that any quotes would be assigned pseudonyms. Throughout the study we paid particular attention to the sensitivity of the subject and to ensuring the study participants’ need for privacy and anonymity.

## Results

In the following, we describe the distinctive needs of AYAs affected by cancer in terms of their social reintegration and return to everyday life during and following treatment. As previously described, these findings are based on analyses of sub-studies 1–4, and they form the background and rationale behind the intervention, which we present in the Discussion section.

Across the empirical material, we found that the AYA cancer experience was heterogeneous and individualized, meaning that the needs of AYAs, and whether, how and to what extent they needed support during and after cancer treatment differed according to 1) their diagnosis and treatment, 2) their form of education and 3) their age, financial challenges and legal entitlements.

### Diagnosis and treatment

The challenges that AYAs experienced in the course of their illness and aftercare differed depending on their diagnosis and type of treatment. These were important determinants of how and whether AYAs experienced late complications, the degree to which they needed help during illness and treatment, and the time spent away from their educational institution. Some AYAs underwent extensive and intrusive treatment and were hospitalized for months or even years—sometimes in isolation—while others completed their treatment within 3–4 months without having to make extensive changes in their everyday lives. The following interview excerpt summarizes these diagnosis-specific challenges:

There are definitely some issues related to the diagnosis. They can be very different – for example, maybe you have testicular cancer, and have to have a serious operation. I don’t even get a chance to greet young patients who are on a surgical ward. They’ve moved on in life before I can meet them. Then there are young people with, for example, acute lymphocytic leukaemia, who have a close relationship with the hospital for a period of two and a half years. There are young people who have bone marrow transplants, are isolated for long periods, and have some very specific problems; they’re in isolation wards and can’t go out into the world. They have different needs from other young people. There are young people with breast cancer, where there are issues about their appearance changing. There are many points along the way that young people have to make decisions about when it comes to reconstruction. […] So there are things that […] are common to all the young people, and then there are individual things they have to deal with; but there are also issues related to the diagnosis itself. (Nurse and youth coordinator)

Thus, it was essential to include the illness characteristics, the accompanying treatment pathway and late complications when considering the potential challenges and needs of AYAs with cancer in their return to everyday life after treatment.

### Type of education and balancing educational requirements and cancer treatment

When receiving the diagnosis, the starting points of the AYAs interviewed differed widely, depending on whether they attended primary school, upper secondary school, higher education or were in transition from one educational level to the next.

The empirical material showed up the complications and complexities of balancing AYAs’ three main priorities: 1) attending to their education or training, 2) attending to their social lives, and 3) attending to their cancer treatment and their health condition. Both practitioners and AYA informants described this balancing and prioritization as extremely difficult. Many AYAs in upper secondary school in particular who are diagnosed with cancer are left in limbo when it comes to attending both to their education and their cancer treatment. This is due to an institutional requirement that the student has to be present at school 90% of the time. Most AYAs attending life-saving cancer treatment are absent from upper secondary school more than 10% of the time and are not able to get their illness-related absenteeism approved or credited. Both practitioners and AYA informants emphasised the stress provoked by these challenges for AYAs with critical illnesses:

There’s a huge issue there, which is absenteeism. […] Something that’s very measurable has become very important. How often they’re present turns out to be extremely important. [...] If they have more than, I think it’s 10% ... [there’s] a limit, then they have to go to an interview with an absenteeism officer – that’s an actual job! […] So, then there’s a whole system that kicks into gear, which makes our young people feel they’re in a vulnerable situation time and time again. And then the counterargument to them is that there’s no need to change it. “Well, they don’t expel them. They don’t expel someone who’s very ill.” But it is extremely stressful for them, and they almost feel like they’re only being allowed to go to school out of charity. (Psychologist)

Furthermore, the empirical material revealed an arbitrary management of support for AYAs with cancer in educational institutions. Some AYAs were met with flexibility, consideration and help, for example by being granted extra time for exams. Others encountered an inflexible, rigid educational system that did not accept absence above 10%. This variation can be related to the fact that AYAs with cancer are ‘rare fish’ as one practitioner put it. In Denmark, the educational system has no fixed procedures, stipulations or requirements to follow in the support of AYAs with critical illness. Consequently, student counsellors in educational institutions may be unsure and unaware of how to deal with the challenges that AYAs experience. In addition, in Denmark, local governments are responsible for education, whereas regions are responsible for the treatment of illness. Often, AYAs themselves must act as intermediaries between these two systems that frame the two central elements of their lives as students and cancer patients. Consequently, “everyone has to reinvent the wheel every time,” as one nurse put it. These findings regarding the educational challenges and absences to which AYAs are subject point to a widespread need among AYAs for consistent support to ensure stable contact and coordination between the educational institution and the hospital during their illness and care pathways. Also, the findings suggest the need for cross-sectional work to help AYAs navigate these systems, local government, the healthcare system and the educational system.

### Age, financial challenges and legal entitlements

The age of AYAs, their financial situation and their legal entitlements were additional determining factors for their needs in terms of returning to everyday life after treatment. Turning 18, and thus reaching the legal age, posed a particular challenge for most AYAs. Those diagnosed with cancer before the age of 18 were usually admitted to the paediatric unit, and their parents were granted paid compassionate leave. In the transition from the paediatric unit to an adult unit, the role of parents changed. They were not as closely involved in treatment as previously, and they were no longer granted paid compassionate leave. According to the practitioners, AYAs with cancer need help and support if they are to take responsibility for their treatment in this transition period.

Turning 18 also marked a distinction in AYAs’ financial situation and their rights and legal entitlements. Practitioners said that most AYAs with cancer, and especially AYAs around the age of 18, need support to navigate a legislative “jungle of paragraphs”, meaning that they need help clarifying what rights and financial support they are eligible for and what is required from a legal viewpoint of AYAs under and over 18, respectively. Practitioners also described the financial effect of illness on some AYAs. In the short term, late complications caused by the illness and treatment may hinder AYAs from receiving the state education grant due to their absence from their course or to their inability to get or keep a student job. In the longer term, absence from work because of late complications may lead to a reduction in pension savings. Also, in the longer term, AYAs who are not members of a trade union or an unemployment fund risk not being insured against critical illness. Financial support for cancer patients does exist; but, as one young informant said, “you have to be well to be ill”, referring to the forms that need to be filled in to receive public subsidies and private grants:

If you don’t manage your sickness allowance yourself, if you don’t know all these things and rules, or you don’t read the fine print on some of the documents – then you either lose some money or some opportunities. (AYA cancer patient)

Despite recent improvements in the availability of information about support opportunities for AYAs with cancer (in 2017 the Danish Cancer Society published a youth cancer manual summarizing relevant knowledge from, among others, healthcare professionals, social workers, psychologists and sexologists), our empirical material shows that the main responsibility for dealing with these practical issues still rests with the AYAs themselves. In the midst of the cancer experience, then, AYAs still have to acquaint themselves with the relevant official legislation and their educational rights and opportunities while coping with sometimes complex financial challenges. Importantly, they are themselves responsible for complying with the legislation in practice, for example by applying for public subsidies and support from private funds, and knowing what forms to fill out, how and when. It is clear that AYAs with cancer typically need help to tackle these financial and legal challenges during and after treatment.

Overall, these findings point to a need among AYAs with cancer for a multidisciplinary intervention accommodating the wide range of opportunities, requirements and challenges presented by a return to everyday life during and after active cancer treatment. In the following discussion, we outline an intervention to meet these needs and discuss our findings and the proposed intervention in relation to other findings in this area of research.

## Discussion

In accordance with other studies, our results show that the transfer period from cancer patient to cancer survivor is a vulnerable period where support and attention from the healthcare system is needed [35, 36]. We recommend the establishment of a multidisciplinary team or professional support group for AYAs completing their treatment for cancer, a team that can work together to address and support the medical and psychosocial problems after concluded treatment. In a recent narrative review, structured communication and coordination between the hospital, home and educational institution has been found to augment school reintegration [35]. Documenting the vocational, educational and financial needs of AYAs with cancer, Fardell, Wakefield, Patterson et al. [35] propose that such communication start at diagnosis and continue after treatment. Therefore, based on the needs and challenges described in this study, we recommend an intervention as a care pathway in which a multidisciplinary team provides individually tailored support for AYAs with cancer, starting from diagnosis and continuing during and after active cancer treatment. The multidisciplinary team we propose extends the recommended multidisciplinary approach described by Ferrari et al. [24] by providing care after active treatment. The team’s main task is to balance educational requirements and cancer treatment with the aim of maintaining AYAs’ social competences and easing their return to everyday life after serious illness. The identified need for differentiation constitutes the basis for the development of the intervention, which allows for a high degree of local adaptation, flexibility and variation [33].

### Social reintegration of AYA cancer – an intervention

**Fig. 2:**
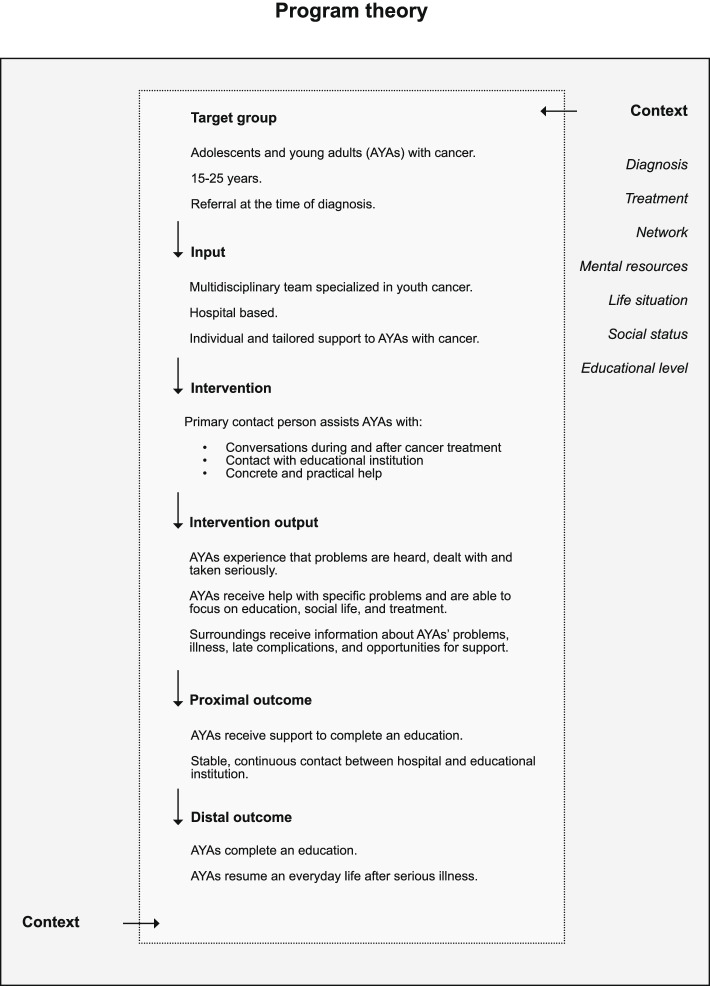
Program theory for the intervention "Social reintegration of AYA cancer"

The central pillar of the intervention is made up of a multidisciplinary team acting both as *coordinators*, building bridges between the healthcare system, local government and the educational system, and supporting AYAs as they navigate within these systems, and as *competent, professional interlocutors*, helping AYAs to tackle and alleviate the physical, bodily, emotional, psychological and social late complications of the illness and treatment (see Fig. 2).

#### Core components of the intervention

Through this intervention, we propose that AYAs with cancer be referred to the multidisciplinary team at the time of diagnosis. The team will constitute an inherent part of the national, standardized cancer patient pathway programme running in parallel to their treatment and will not be a voluntary service that AYAs are obliged to discover by themselves. The multidisciplinary support team will be situated at the treating hospital and be available, starting from the time of diagnosis and continuing during and after treatment. Their main task will be to ensure AYAs’ social reintegration to everyday life during and beyond active treatment. The team will include a range of professional competencies to ensure broad coverage of AYAs’ needs. First, professional competencies in healthcare and knowledge of different forms of cancer, different types of treatment as well as the associated late complications are crucial components of the team. This role could be fulfilled by an oncological nurse. Second, the team must include professional understanding of the educational system (e.g. a student counsellor or a teacher) to assist AYAs in planning and administering their courses of study during and after the illness period. Third, it will need the skills of a social worker to help AYAs to deal with financial issues and with local government administration. In addition, according to all informants (practitioners as well as AYAs), other professional competencies, such as a psychologist, psychiatrist and a gynaecologist, might be required in the multidisciplinary team. Importantly, all professionals within the team must undergo specialist training to ensure a full and deep understanding of the AYA population and of the ramifications of youth cancer and youth support. As part of this specialist training, all team members must be able to carry out the HEEADSSS psychosocial interview for adolescents [32].

Once an AYA is diagnosed with cancer, he or she will be assigned a primary contact person from the multidisciplinary support team. Besides handling the primary contact with the AYA, the contact person may both assist the AYA in establishing contact with other team members and communicate professional feedback to and from the other team members.

Specifically, the team will assist AYAs with cancer in relation to:

- *Conversations with AYAs starting from the time of diagnosis and continuing during and after cancer treatment.* During these conversations, the appointed team member will address the physical, bodily, emotional, psychological, social and financial challenges faced by the AYAs. The conversations should be based upon the HEEADSSS interview model and will take place at the hospital, when/if the AYA is hospitalized, and at home, when the AYA has finished treatment. Because late complications are strongly associated with the specific cancer diagnosis and treatment, the competencies of the healthcare professional, e.g. an oncological nurse, will be particularly relevant here.
- *Ensuring continuous, stable contact with the AYA’s educational institution.* Here, the appointed team member will contact the AYA’s educational institution, typically through a student counsellor, to ensure the best possible conditions for the AYA in terms of continuing and completing their education. In collaboration with the AYA and the student counsellor, the team member will identify the wishes and needs of the AYA in terms of continuing their education. In particular, the professional with specialist educational competencies within the team, e.g. a teacher or student counsellor, might be involved here, providing advice, for example, about existing educational support opportunities (both financial and practical support) for AYAs with critical or chronic illnesses. Furthermore, our results indicate the need to pay special attention to youth education programmes and, in particular, upper secondary schools due to AYAs’ experiences of not being able to secure approval for treatment-related absences. By comparison, the results indicate that AYAs’ association with university departments seems to be relatively smooth and unproblematic, partly due to a greater flexibility, and because absences are not usually registered.
- *Providing concrete, practical help to AYAs*, for example, in dealing with financial and legal issues, applying for support from private funds, and contacting and dealing with local government. The competencies of the professional social worker will be particularly relevant here.

Overall, the multidisciplinary team must possess a wide range of knowledge regarding cancer diagnoses and treatment, the educational system, the organization of local government, relevant foundations, patient rights and the lives of young people in general. The tasks of the multidisciplinary team will include dealing with the public sector to accommodate the AYAs’ individual issues.

### Findings in relation to other studies

Our findings indicate the need for an intervention involving a multidisciplinary team that can, on the one hand, assist AYAs with cancer in navigating the administrative, legal, financial and psychological issues that can cause severe stress and, on the other hand, can support their social re-entry into everyday life e.g., through educational and financial aid. Several studies point to recognizable benefits of educational and vocational support for AYAs with cancer [37–39]. Among other things, research conducted by Dax et al. show that the majority of AYA cancer patients who receive educational and vocational services remain in educational and/or employment [37]. However, further research is still needed to understand and evaluate the support AYAs need to achieve social reintegration after active treatment [18, 37].

We suggest, therefore, that the proposed intervention be evaluated in a series of pilot studies testing its feasibility and effectiveness while also determining the form and procedures involved [33]. Before embarking on a full-scale evaluation of these studies, it will be essential to incorporate the responses of AYAs and of the professionals who will be delivering the intervention [37]. Due to a high degree of local adaptation, flexibility and variability in the intervention design, we recommend that the intervention is evaluated following the Medical Research Councils (MRC) guidance for evaluation of complex interventions [33]. Besides rendering the mechanisms of change visible, the programme theory can be applied as a working tool for an evaluation of the intervention [40].

We did not find support for the design of a peer-to-peer intervention, which has been identified in other studies [41]. Instead, the informants in our study expressed a need for support from professionals who have the necessary qualifications and competences to discuss everything from educational problems to late complications. One possible explanation may be that the AYA informants were primarily recruited through AYA cancer support groups run by the Danish Cancer Society, which are widespread in Denmark. Additionally, in some of the larger hospitals in Denmark, where we also recruited informants, there are age-appropriate supportive facilities and activities developed for AYAs with cancer, such as *Kræftværket* [42]. One aim of *Kræftværket* is, indeed, to give AYAs with cancer the chance to connect with peers [42]. Finally, the Danish Cancer Society has developed a peer-to-peer youth cancer manual, in which both AYAs with cancer and various professionals provide information and counselling about such issues as friendship, love, sex, treatment, hospital life, late complications and mental reactions [43]. The manual is handed out to all young cancer patients between the ages of 15 and 39 at the time of diagnosis. This means that the majority of AYAs with cancer in Denmark are offered peer-to-peer support, which may explain why this was not a need found in the empirical material. Instead, our findings point to a need for help with navigating the local government and securing stable contact with the educational institution. A Danish study by Pedersen, Boisen, Midtgaard et al. [44] confirms these findings, stating that AYAs experience difficulties in navigating the complexities of public and educational systems.

### Strengths and limitations

In developing this intervention, we chose a broad target group including all cancer patients between the ages of 15 and 25. This relatively wide AYA age span is also characteristic of other studies and constitutes a general challenge for AYA cancer research and practice [10, 42]. Clearly, the needs, challenges, requirements and general life situation of a 15-year-old and a 25-year-old may be very different. With the strong focus on the multidisciplinary team providing individualized and tailored support, we would argue that the proposed intervention is designed to accommodate and tackle these diverse needs. The emphasis on tailoring may be particularly relevant when intervening in the AYA population. Studies show that AYAs may be difficult to recruit and maintain whether in intervention programmes or in outpatient treatment, indicating that programmes and services targeting AYAs should be organised with a high degree of tailoring and flexibility [45, 46].

We argue that the proposed intervention is well suited to address social inequalities in cancer care and outcomes, as it builds on tailored support and because referral to the multidisciplinary team will be an inherent part of the care pathway and not a voluntary service. Some studies find social variability among the survivors and suggest attention to social inequality on both the societal and individual levels in cancer research [47]. We argue that the flexible design and organisation of the intervention allows the multidisciplinary team to provide additional support to AYAs with cancer who lack practical, emotional and/or social support from their network and in this way can assist their social reintegration after treatment.

This study is based on a sequential design drawing on multiple qualitative methods. This kind of design takes time, both to conduct and analyse. However, despite the longitudinal collection of empirical material, the design adds in-depth and relevant insights into the challenges and needs related to AYAs’ cancer journey.

## Conclusion

Based on the perspectives of both practitioners and AYAs affected by cancer, this study outlines an intervention designed as a care pathway in which a multidisciplinary team provides individual and tailored support to AYAs with cancer, starting from the time of diagnosis and continuing during and after cancer treatment.

Even though the intervention focuses on AYAs with cancer, the findings may have a more general relevance for young people who may need similar forms of support after encountering life-shattering events, such as other serious diseases, accidents or loss of close family members. This wider generalizability not only strengthens the public health relevance and potential of social reintegration services, but also increases the likelihood that such services can be implemented on a wider scale. In particular, we expect that this intervention could be of relevance and appreciated among AYAs with chronic diseases in general.

## Supplementary Information

**Additional file 1.** Interview guide questions for interviews with AYAs who had, or had previously had, cancer. Full set of questions from the interview guide with AYAs who had, or had previously had, cancer.

**Additional file 2.** Interview guide questions for interviews with practitioners working within the field of cancer or AYAs with chronic conditions. Full set of questions from the interview guide with practitioners working within the field of cancer or AYAs with chronic conditions.

## Data Availability

Due to the sensitive nature of the data generated and the possibility of identification of individuals, the data generated and/or analysed during the current study are not publicly available.

## Abbreviations

AYA: Adolescents and young adults
HEEADSSS: Home environment, Education and employment, Eating, peer-related Activities, Drugs, Sexuality, Suicide/depression, and Safety from injury and violence
