# Supplementary material for: Development of an intervention for the social reintegration of adolescents and young adults affected by cancer
Source: BMC Public Health. 2022 Feb 5;22:241. doi: 10.1186/s12889-022-12611-4 (PMC8818212; doi:10.1186/s12889-022-12611-4)
Supplement: Supplementary file 1 — Additional file 1. Interview guide questions for interviews with AYAs who had, or had previously had, cancer. Full set of questions from the interview guide with AYAs who had, or had previously had, cancer. [file 12889_2022_12611_MOESM1_ESM.docx]

**Interview guide for interviews with AYAs who had, or had previously had, cancer.**

Personal information

To begin with, I would like to know:

- your age
- where do you live
- do you live with family / alone / with boyfriend / do you have children?
- are you under education (which) / or do you have a job (which)?

The Course of the disease- and treatment

What kind of cancer diagnosis do / did you have?

When did you get the diagnosis? (month / year)

What happened when you got the diagnosis? Did you receive treatment immediately? What treatment have you received? Are you finished with treatment? When did you stop treatment? (month / year)

Which hospital ward have you been admitted to? Multiple departments at different times? Possibly far away from home?

How did you experience the contact with resp. doctors and nurses?

How did you react when you were diagnosed with cancer? What did you do?

How did your surroundings react? (family, friends, boyfriend, school / classmates, colleagues, teachers / educators, others) - what did they do? What did they say? How did they know you had cancer? Were there any reactions that you particularly noticed?

How do you think being young with cancer is different from being an adult with cancer, for example? Is there anything that is particularly difficult about being young with cancer?

Were you still able to attend school / study / work when you received treatment? How did you do it?

The course after treatment / return to everyday life

How did you experience ending treatment?

Did you feel that you were finished being treated? Do you still feel ill?

How did you feel?

How did you react? What did you do? Did you feel?

How did your family / friends / boyfriend react? What did they do? What did they say?

How did you experience going back to everyday life and to your work? How would you describe that time / period?

What were you most looking forward to? What were you least looking forward to?

Has your everyday life changed a lot after you have finished treatment?

In what way has your illness and treatment affected your everyday life?

What is the biggest difference between your everyday life before and after you get your disease?

Were there any activities / things that you could not do during the treatment that you resumed?

Were there activities / things you could not do even though the treatment was over? As you wanted to do?

Do you think that your illness limits you in doing things - or vice versa: has the illness made you do some other things than you did before you became ill?

Do you need (additional) help to get better in everyday life? What help might be right for you?

Would you even call it 'going back to everyday'? Do you feel like you should go back to your old life? Or is it a new life that has started after your illness

Side effects

Did you experience any side effects as a result of the disease or treatment?

Physical? (visible as e.g. scars; invisible as fatigue, lack of endurance ..)

Mental / mood? (anxiety; depression; fear of getting sick again…)

Has your body changed appearance (e.g. hair loss, weight loss ..)? How do you feel about it? How did your family / friends / boyfriend react to it?

Have some of the side effects made it difficult to cope with everyday life? Can you describe a day that is difficult to get through - how do you feel? what do you do; is there anyone who can help you?

Did any doctors / nurses tell you about any side effects / late effects that could occur after the treatment was completed?

Has the disease affected your ability to have children? Are you worried about having children?

Do you feel that you were given the information you needed by doctors and nurses about how the disease can affect the possibilities of having children?

Network:

How did the course of the disease affect your relationship with resp. your parents, siblings, friends, boyfriend, others?

Positive and negative?

Who from your network has had the greatest impact on you after you became ill?

Has the course of the disease meant that your circle of friends has changed? How?

Have you made new friends as a result of the disease? For example, in the Young Cancer group?

Do you participate in the Young Cancer group events? Why / why not? What does it give you? Do you think that it required overcoming / a special courage to show up in the Young Cancer group?

How long have you been in the Young Cancer group? Do you think that at some point you will no longer be part of that community?

Are there any communities / people you have withdrawn from after you became ill? Why?

Social media:

Do you use facebook, instagram, snapchat, blogs / other social media?

Have you used social media to tell about your illness? Try to describe how…

What does it add to you when you use social media to tell about your illness?

Need for support and help during the course of treatment and after treatment

In the course you have described, what specific things or activities have you needed help to do?

Did you receive that help? Who helped you? Try to describe situations where someone has done something good.

Who else in your social circle has supported you? What have they done to help you? What did this help mean to you? Try to describe / give ex…

- including: parents, siblings, friends, boyfriend, school / classmates, colleagues, other cancer patients (young people)

- try to describe who you use for what?

- what is it that your mother / friend / boyfriend etc. does  try to give ex.

Who are you talking to about having cancer? (family, friends, teachers, others with cancer, others? Psychologist?). What are you talking about? Can you give an example?

Are there any places / situations where you do not want to talk about cancer? Are there any places / situations where you wonder if they are not talking to you about your cancer?

Does anyone need you to support them? Are you able to support them?

Is there anything or anyone that you think could have helped you during the period when you finished treatment and had to return to everyday life? Something we have not talked about?

What types of help / support were you missing?

Return to work:

How was it to return to your job (after diagnosis or after treatment)?

Did you feel any different? In what way, try to describe…

How did your colleagues react?

Have you had contact with your colleagues all the time during your illness?

Do you feel that your colleagues understood what you were going through? How did it manifest itself?

Has the disease affected your work? How? (e.g. need for more breaks ..)

Were there certain people who helped you return to work (e.g. colleagues, hospital staff, other young people with cancer, social workers, psychologist)?

Is there anything or anyone that you think could have helped you during the period when you finished treatment and had to return to work?

What types of help / support do you think is missing?

The future and plans for life

Has something positive also come out of you being ill? Has it strengthened you in any way? Have you changed your view of what is important in life? Try to describe…

Have you given any thought to how illness and treatment will affect your everyday life in the future?

What other thoughts do you have about the future? When you think about the future - what kind of thoughts do you have? If it's a little difficult - try to say a little about what you dream about? What would you like your life to look like?

So let's finally do a thought experiment, because even though I've asked a lot of questions, you may well be thinking - why are they not asking about it - so: If you were in my place - and should learn something , about what it is like to be young and get a life-threatening illness like cancer. So are there any particular questions you would like to ask? Or places you would go because you think you could really learn something?

Do you know anyone who would like to talk to us?

Thank you so much for participating
