# Supplementary material for: Development of an intervention for the social reintegration of adolescents and young adults affected by cancer
Source: BMC Public Health. 2022 Feb 5;22:241. doi: 10.1186/s12889-022-12611-4 (PMC8818212; doi:10.1186/s12889-022-12611-4)
Supplement: Supplementary file 2 — Additional file 2. Interview guide questions for interviews with practitioners working within the field of cancer or AYAs with chronic conditions. Full set of questions from the interview guide with practitioners working within the field of cancer or AYAs with chronic conditions. [file 12889_2022_12611_MOESM2_ESM.docx]

**Interviewguide for interviews with practitioners working within the field of cancer or AYAs with chronic conditions**

Information about the paractitioner:

Initially, I would like to know a little more about your work. Can you tell me about your work?

- How do you meet the young people? When? How often / for how long?
- How many young people do you see for check-ups / treatment?
- How do you experience problems around young people and cancer?

The target group (AYAs with cancer aged 15-30):

- In your opinion, what are the main challenges young people face?
- How you think being a young person with cancer is different from being an adult with cancer? Is there anything that is particularly difficult about being young and seriously ill?

### Reintegration:

- In you experience, what do AYAs with cancer need after finishing treatment? / Which challenges do young people have after finishing treatment and or hospitalization?
- In what way can young people with serious illness be helped after they have finished treatment?
- Through interviews with young people with cancer, we find that there are some practical (financial), mental (low self-esteem and loneliness) and physical (fatigue and changed appearance) barriers / challenges that affect their everyday life after treatment. What do you experience as the biggest challenge for the young people?
- If you all resources in terms of finances and otherwise structures such as health care, social services and education, how would you organize the ideal 'course after cancer / serious illness' for young people?

### Education:

- What is the biggest challenge for young people with cancer who have to return to education and everyday life after finishing treatment? (Why)
- (Do some groups of young people have more difficulties than others? (Eg vocational school children vs. gym young people or…?))
- Can you tell about a young person who has had difficulty resuming education and everyday life after finishing treatment? (why do you think he / she had a particularly difficult time?)
- Can you tell about a young person where it has been easy / easier to resume education and everyday life after finishing treatment? (what do you think made it easier for him / her?)
- One of the things we hear in our interviews of young people who have or have had cancer is that it can be difficult to get back to school / education / etc for many different reasons. What advice would you give to a young person with a serious illness who, after completing treatment, should resume an educational course? (why is it important? - are there other things that could be important)
- What advice would you give a study counselor / teacher who should receive / help a young person with a serious illness after finishing treatment back in the educational process? (why is it important? - are there other things that could be important)

### Future initiatives:

- Which approaches should be included in an effort to help and support young people with cancer to resume a normal youth life after completing cancer treatment, how would you approach such a task? What elements / areas should such an intervention focus on? (Why is it important?)
- - (How would you design an intervention to support the target group to resume or complete a youth education?)

**Coordinator function: our initial thoughts on an intervention…**

### Finishing the interview:

- Are there any things you think we need to ask? Do you have something you think is important to add about young people with serious illness? (An overview of offers for young people with cancer in general?)

- Do you have suggestions for where we can seek further information (projects, enthusiasts, etc.?). Do you know anyone who wants to talk to us?

- Is there anything special you would like to bring forward / focus on?

Finally, I want to hear from you if I can contact you by phone for any follow-up questions / ambiguities?

Thank you so much for participating!
